# Loss of participation among evacuees aged 20–37 years in the disaster cohort study after the Great East Japan Earthquake

**DOI:** 10.1038/s41598-022-23896-1

**Published:** 2022-11-15

**Authors:** Kana Yamamoto, Morihito Takita, Masahiro Kami, Yuta Tani, Chika Yamamoto, Tianchen Zhao, Tetsuya Ohira, Masaharu Maeda, Seiji Yasumura, Akira Sakai, Mitsuaki Hosoya, Kanako Okazaki, Hirooki Yabe, Masaharu Tsubokura, Michio Shimabukuro, Hitoshi Ohto, Kenji Kamiya

**Affiliations:** 1 Department of Radiation Health Management, Fukushima Medical University, Fukushima, Fukushima 960-1295 Japan; 2 Medical Governance Research Institute, 2-12-13 -201 Takanawa, Minato, Tokyo, 108-0074 Japan; 3 Radiation Medical Science Center for the Fukushima Health Management Survey, Fukushima Medical University, Fukushima, Fukushima 960-1295 Japan; 4 Department of Epidemiology, Fukushima Medical University School of Medicine, Fukushima, Fukushima 960-1295 Japan; 5 Department of Disaster Psychology, Fukushima Medical University School of Medicine, Fukushima, Fukushima 960-1295 Japan; 6 Department of Public Health, Fukushima Medical University School of Medicine, Fukushima, Fukushima 960-1295 Japan; 7 Department of Radiation Life Sciences, Fukushima Medical University School of Medicine, Fukushima, Fukushima 960-1295 Japan; 8 Department of Pediatrics, Fukushima Medical University School of Medicine, Fukushima, Fukushima 960-1295 Japan; 9 Department of Physical Therapy, Fukushima Medical University School of Health Sciences, Fukushima, Fukushima 960-8516 Japan; 10 Department of Neuropsychiatry, Fukushima Medical University School of Medicine, Fukushima, Fukushima 960-1295 Japan; 11 Department of Diabetes, Endocrinology and Metabolism School of Medicine, Fukushima Medical University School of Medicine, Fukushima, Fukushima 960-1295 Japan; 12 Research Institute for Radiation Biology and Medicine, Hiroshima University, Hiroshima, 734-8553 Japan

**Keywords:** Natural hazards, Health care

## Abstract

The present study aimed to clarify the characteristics of young evacuees who had missed the Comprehensive Health Check of the Fukushima Health Management Survey (FHMS) after the Great East Japan Earthquake in 2011. The FHMS has been conducted as a prospective cohort study to evaluate the health status of evacuees annually after the great earthquake in 2011. This study focused on the annual participation rate in the Comprehensive Health Check of evacuees aged between 20 and 37 years in 2011 who evacuated due to the Fukushima Daiichi Nuclear Power Plant accident. The characteristics of subjects who did not participate after the second survey year were identified with a multivariate logistic regression model. The participation rate was estimated at 26.6% (9720 among 36,502 residents) and 15.6% (5691 residents) in 2011 and 2012, respectively. The logistic regression model revealed the following characteristics at baseline as independent predictors of non-participation after the second year of the survey: age ≤ 24 years (adjusted odds ratio 2.11, 95% CI 1.84–2.42), 25–29 years of age (1.28, 1.13–1.45), men (1.52, 1.38–1.69), evacuation outside the municipality but within Fukushima prefecture (1.54, 1.40–1.70), evacuation outside the Fukushima prefecture (1.40, 1.21–1.63), anemia (1.23, 1.06–1.43), smoking habit (1.34, 1.21–1.48), and drinking habit (1.20, 1.09–1.32). A medical history of heart disease showed opposite odds ratios, which indicate the association with continuous participation (0.43, 0.26–0.72, respectively). We observed deteriorated participation in the prospective study of the Comprehensive Health Check of the FHMS among evacuees of a younger age group, men, those evacuated outside their municipalities, and those with history of anemia, smoking and drinking habits. Hence, the cohort study may have missed certain population groups with worse health behaviors. Thus, it is necessary to consider various measures to increase the participation rate in the disaster cohort study to understand the long-term health effects of disasters on younger residents in evacuation zones.

## Introduction

The Great East Japan Earthquake in 2011 was a *triple* disaster cascading the earthquakes, the huge tsunami, and the nuclear accident at the Fukushima Daiichi Nuclear Power Plant (FDNPP). Natural disasters themselves cause medical conditions such as injuries, exposure to harmful substances, diseases, and mental health issues^1^. Delays and barriers in access to medical care indirectly affect human health^2^. The radiation disaster, such as the nuclear accident of Chornobyl and the atomic bombing of Hiroshima and Nagasaki, which are artificial disasters, could also cause direct and indirect health issues^3,4^. The current Russia–Ukraine war poses unpredictable radiation hazards in areas surrounding nuclear power plants^5^. Analyzing past disaster cohorts is essential when considering health measures for future disasters.

It is critical for medical professionals to identify and protect populations vulnerable to disasters. Post-disaster cohort studies have been published for Hurricane Maria^6^, Hurricane Katrina^7^, Red river flood^8^, Sichuan Earthquake^9^, Fort McMurray Wildfire^10^, and Sumatra Earthquake^11^. These studies have consistently shown that the elderly, poor, and structurally disadvantaged populations are more vulnerable to the indirect effects of natural disasters. We experienced a consistent and statistically significant increase in extreme weather conditions and, in turn, natural disasters during the past 30 years, from 1990 to 2020^12^. However, only a few of them are longitudinal studies, and most are small in size. In disaster cohorts, evacuees move to unfamiliar locations from their original residence, making it difficult to track their health status in the long term^13^. Therefore, the long-term effects of disasters remain unclear.

In response to the Great East Japan Earthquake, the Fukushima prefectural government launched the Fukushima Health Management Survey (FHMS) in June 2011 to investigate the health status of residents and utilize the data obtained for health promotion, with a target population of approximately 2.05 million^14^. This large-scale prospective cohort study attempted to identify the influence of extended evacuation by reviewing the health information of evacuees, assessing the prevalence of various diseases, and promoting their health. The Comprehensive Health Check, in addition to the Basic Survey of FHMS, has been conducted especially for the residents of all age groups living in evacuation zones designated by the national government because of the FDNPP accident between March 11, 2011, and April 1, 2012. The Comprehensive Health Check evaluates complete blood cell counts with leukocyte fractions, urine occult blood, serum creatinine concentration, estimated glomerular filtration rate, and uric acid in conjunction with regular medical checkups in Japan^14^.

The FHMS is one of the largest prospective disaster cohort studies to date. The FHMS is a census survey that includes all evacuees and was initiated by the Fukushima prefectural government with a funding of approximately 84 billion yen. The national government of Japan financially supports the FHMS. The prefectural government commissioned Fukushima Medical University to conduct the FHMS. It has revealed disaster-related health risks in residents forced to evacuate for extended periods of time. Because of the evacuation and subsequent lifestyle changes, an increased risk of worsening metabolic diseases such as obesity, dyslipidemia, and diabetes has been observed^15–17^. The prevalence of diabetes mellitus significantly increased after the disaster, and its incidence was significantly greater among evacuees than non-evacuees^16^. Blood pressure also increased among the victims, especially evacuees, after the Great East Japan Earthquake. The evacuation itself might be associated with an increased risk of hypertension among men^18^. The survey showed the effects of the disaster on dietary habits: the overall intake of meat and fish among young people decreased after the disaster^19^. However, the coverage rate of the Comprehensive Health Check of the FHMS has been low, ranging between 20.2 and 30.9% of the total population, despite continuous efforts to promote the study^20,21^. The characteristics of victims who missed the FHMS have not been sufficiently investigated yet.

We recently analyzed 8 years of data on young evacuees aged between 20 and 44 years who underwent the Comprehensive Health Check of the FHMS and investigated the impact of the Great East Japan Earthquake on anemia^22^. In the investigation, we found an increase in the number of young evacuees who did not participate in the Comprehensive Health Check during the study period. In this study, we specifically determined the characteristics of young evacuees who had not participated in the Comprehensive Health Check of the FHMS after the second year in this study as an exploratory analysis of the previous report^22^. The findings of this study suggest ways to improve the coverage of the Comprehensive Health Check and, in turn, contribute to promoting the health status of young evacuees.

## Materials and methods

### Study design and procedure

The FHMS is a prospective cohort study which initiated by the Fukushima Prefecture in July 2011 after the Great East Japan Earthquake. The FHMS was prospectively designed for all residents of Fukushima Prefecture to assess the prevalence of diseases and promote their health status. The Comprehensive Health Check, as a part of the FHMS, was intended for those who lived in a nationally designated evacuation area because of the FDNPP accident between March 11, 2011, and April 1, 2011 (Fig. 1 in the Supplement). This study was designed as an exploratory analysis with the FHMS database.

### Participants

The subjects of the Comprehensive Health Check were notified annually by mail based on the resident registry of municipalities. After the notification, they receive the Comprehensive Health Check at Fukushima Medical University or individually at designated medical institutions. Even if they move out from the Fukushima prefecture, they can receive the Comprehensive Health Check outside the prefecture by notifying their change of address. Without notification of address change, however, it will be no chance for the eligible individuals to know the opportunity of the Comprehensive Health Check.

Our previous study on anemia in reproductive-age women focused on females aged between 20 and 44 who had participated in the Comprehensive Health Check in FHMS in the 2011 to 2018 survey, where a substantial decrease in the participation rate was observed as the study progressed^22^. This study targeted the participants of the Comprehensive Health Check aged between 20 and 37 years in 2011, the first survey year, to determine their participation for an 8-year follow-up period as an exploratory analysis of the previous study. Of note, the survey year in 2011 started in July 2011 and ended in March 2012. In the following years, data by March every year were summarized.

### Ethical considerations

The institutional review board of Fukushima Medical University approved the FHMS, including exploratory analyses (approval numbers: 1319 for the Comprehensive Health Check, 2020239 for the mental and lifestyle survey, and 29064 for a bridge between the Comprehensive Health Check and mental-lifestyle survey). Written informed consent was obtained from all participants prior to the survey. This study was performed in accordance with the Ethical Guidelines for Medical and Health Research Involving Human Subjects in Japan.

### Measures

The primary endpoint of this study is the annual participation status of the Comprehensive Health Check. Secondarily, we assessed the characteristics associated with non-participation after the second survey year of 2012 by utilizing the participant variables in 2011 as a baseline. The survey assessed their evacuation status, body mass index (BMI), blood pressure, medical history, smoking history, alcohol habit, and lipid and glycemic metabolism data.

The evacuation status was classified into the following three groups by comparing the address information on the municipalities between resident registry at pre-quake of March 2011 and at the post-quake initial survey of 2011: evacuation within the same municipality, outside municipality but within Fukushima Prefecture, and outside of the Fukushima Prefecture. The medical history, smoking history, and alcohol habits were captured from the self-reported questionnaire. Participants’ medical history indicated concomitant medical conditions. Smoking history was denoted when participants reported current or past smoking habits. Alcohol habit was indicated by participants’ responses on a four-point Likert scale, ranging from drinking every day to drinking occasionally.

### Statistical analysis

The participation rate of the Comprehensive Health Check at the baseline was assessed with the number of participants in the initial survey year divided by the estimated target population in March 2011. The participant characteristics were summarized with descriptive statistics. We classified the study cohort into two groups depending on their participation status: those who did not participate after the second survey and those who attended at least once (the non-participation cohort and the control, respectively). Two-group comparisons were performed using independent t-tests and Pearson’s chi-square tests on continuous and categorical data. We employed multivariate logistic regression analysis to predict the likelihood of missed participation in the Comprehensive Health Check after the second survey year of 2012 for characteristics in the first year of 2011. The explanatory variables include the baseline characteristics of age, gender, BMI, evacuation status, disease histories of hypertension, cerebrovascular disease, heart disease, anemia, diabetes mellitus, dyslipidemia, and kidney disease. We excluded the systolic and diastolic blood pressures, laboratory outcomes of hemoglobin A1c, low-density lipoprotein (LDL) cholesterol, and triglyceride from the multivariate analysis because of colinearities expected with the disease history of hypertension, diabetes mellitus, and dyslipidemia. All statistical analyses were performed using a two-sided approach, and statistical significance was considered when the *p* value was less than 0.05. Statistical evaluation was performed using the IBM SPSS Statistics version 28 (Armonk, NY, USA).

## Results

### Coverage of the Comprehensive Health Check in the younger population in the 2011 survey

**Figure 1:**
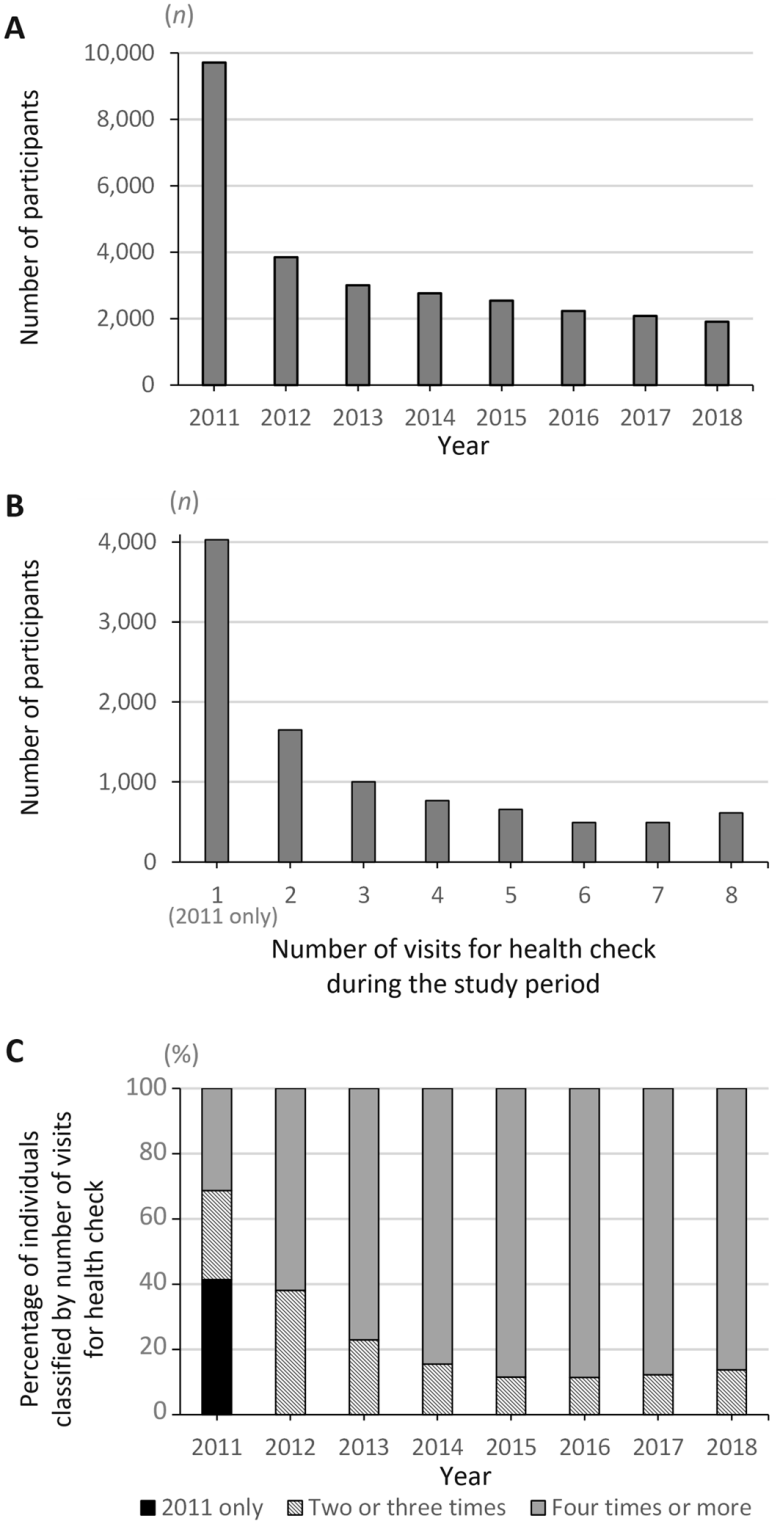
Annual participation of young evacuees in the Comprehensive Health Check of the Fukushima Health Management Survey. The annual change in participation in the Comprehensive Health Check of the Fukushima Health Management Survey (FHMS) is shown. The number of participants was classified according to year (**A**). The number of individual visits during the study period between 2011 and 2018 was counted (**B**). The annual percentage of participants is shown after classifying by the number of visits during the study period (**C**).

The target population aged between 20 and 37 years and eligible for the Comprehensive Health Check in the FHMS in March 2011 was estimated as 36,502 persons (eTable 1 in the Supplement). A total of 9720 evacuees (4029 men and 5691 women) participated in the Comprehensive Health Check in 2011. Thus, the participation rate in the initial year of the Comprehensive Health Check was 26.6%. On the other hand, 5691 persons (15.6%) attended the Comprehensive Health Check once or more after the second survey year of 2012 (Fig. 1).

The median number [interquartile range] of visits for Comprehensive Health Check was 2^1–4^ during the survey years 2011–2018. Four thousand twenty-nine evacuees, accounting for 41% of the 2011 survey participants, did not attend the Comprehensive Health Check after 2012. In contrast, 80% or more of the participants after the 2014 survey visited four times or more during the study period. The proportion of men declined by approximately 10% after 2014 compared to 2011 (Fig. 2 in the Supplement). The proportion of those aged between 20 and 25 years decreased to approximately 15% after 2013 compared to 2011.

### Univariate assessment on the characteristics associated with non-participation after the second survey

We compared the characteristics of participants who did not attend the Comprehensive Health Check after the 2012 survey (non-participation cohort) with those who participated at least once in 2012 or in the following years (Table 1). The non-participation cohort was significantly younger, with more men, higher BMI, and higher blood pressure when compared to the control (average [standard deviation] or percentage: 29.3 [5.2] and 30.6 [4.8] years old; *p* < 0.001, 47% and 33% men; *p* < 0.001, 23.0 [4.5] and 22.6 [4.4] kg/m^2^ BMI; *p* < 0.001, and 115 [14] and 114 [14] mmHg systolic blood pressure; *p* < 0.001, in non-participation cohort and the control, respectively). Significant differences were also observed in the evacuation status (*p* < 0.001).

**Table 1:** Comparison of study participants after classifying participation status after the 2012 survey.

Variables in 2011 survey	Participation in health checkups after 2012		*p* value
	No	Yes	
	(*n* = 4029)	(*n* = 5691)	
**Age; *n* (%)**			< 0.001
≤ 24 years	921 (23)	791 (14)	
25–29 years	1041 (26)	1401 (25)	
30–33 years	961 (24)	1546 (27)	
≥ 34 years	1106 (26)	1953 (34)	
**Gender—men; *n* (%)**	1879 (47)	1877 (33)	< 0.001
**Body mass index (kg/m**^**2**^**)**			< 0.001
< 18.5	407 (10)	747 (13)	
18.5–22.9	1938 (48)	2801 (49)	
23.0–24.9	658 (16)	818 (14)	
25.0–29.9	696 (17)	920 (16)	
≥ 30.0	330 (8)	405 (7)	
**Systolic blood pressure (mmHg)**	115 (14)	114 (14)	< 0.001
**Diastolic blood pressure (mmHg)**	70 (11)	69 (11)	0.006
**Evacuation; *n* (%)**			< 0.001
Within municipality	1260 (31)	2211 (39)	
Outside municipality but within Fukushima prefecture	2023 (50)	2395 (42)	
Outside Fukushima prefecture	746 (19)	1085 (19)	
**Disease history; *n* (%)**			
Hypertension	42 (1.1)	50 (0.9)	0.46
Cerebrovascular disease	12 (0.3)	11 (0.2)	0.4
Heart disease	26 (0.7)	65 (1.2)	0.01
Anemia	422 (10.7)	645 (11.7)	0.12
Diabetes mellitus	20 (0.5)	31 (0.6)	0.78
Dyslipidemia	21 (0.5)	46 (0.8)	0.11
Kidney disease	59 (1.7)	85 (1.7)	0.8
**Smoking history; *n* (%)**	1512 (38.2)	1629 (29.4)	< 0.001
**Drinking habit; *n* (%)**	2019 (51.0)	2460 (44.3)	< 0.001
**Laboratory results on metabolic outcomes**			
Hemoglobin A1c (%)	5.1 (0.6)	5.1 (0.5)	0.54
LDL-C (mg/dL)	112 (31)	112 (30)	0.86
Triglyceride (mg/dL)	91 (75)	89 (72)	0.22

The numbers (percentages of non-participation or control groups) are shown. Two-group comparisons between the non-participation cohort and the control were performed with the independent t-tests and Pearson’s chi-square tests on continuous and categorical data, respectively.

*LDL-C* low-density lipoprotein cholesterol.

No significant differences in medical history were detected between the two groups, except a history of heart disease, which was significantly lower in the non-participation cohort (0.7% and 1.2% in the non-participation and control groups, respectively; *p* = 0.01).

Significantly higher proportions were seen for smoking history and drinking habit in the non-participation cohort (*p* for smoking history < 0.001; 38.2% and 29.4%, and *p* for drinking habit < 0.001; 51.0% and 44.3%, in the non-participation cohort and the others, respectively). No significant differences were found in the metabolic markers of hemoglobin A1c, low-density lipoprotein (LDL) cholesterol, and triglyceride levels between the two groups.

### Baseline characteristics associated with non-participation cohort

**Figure 2:**
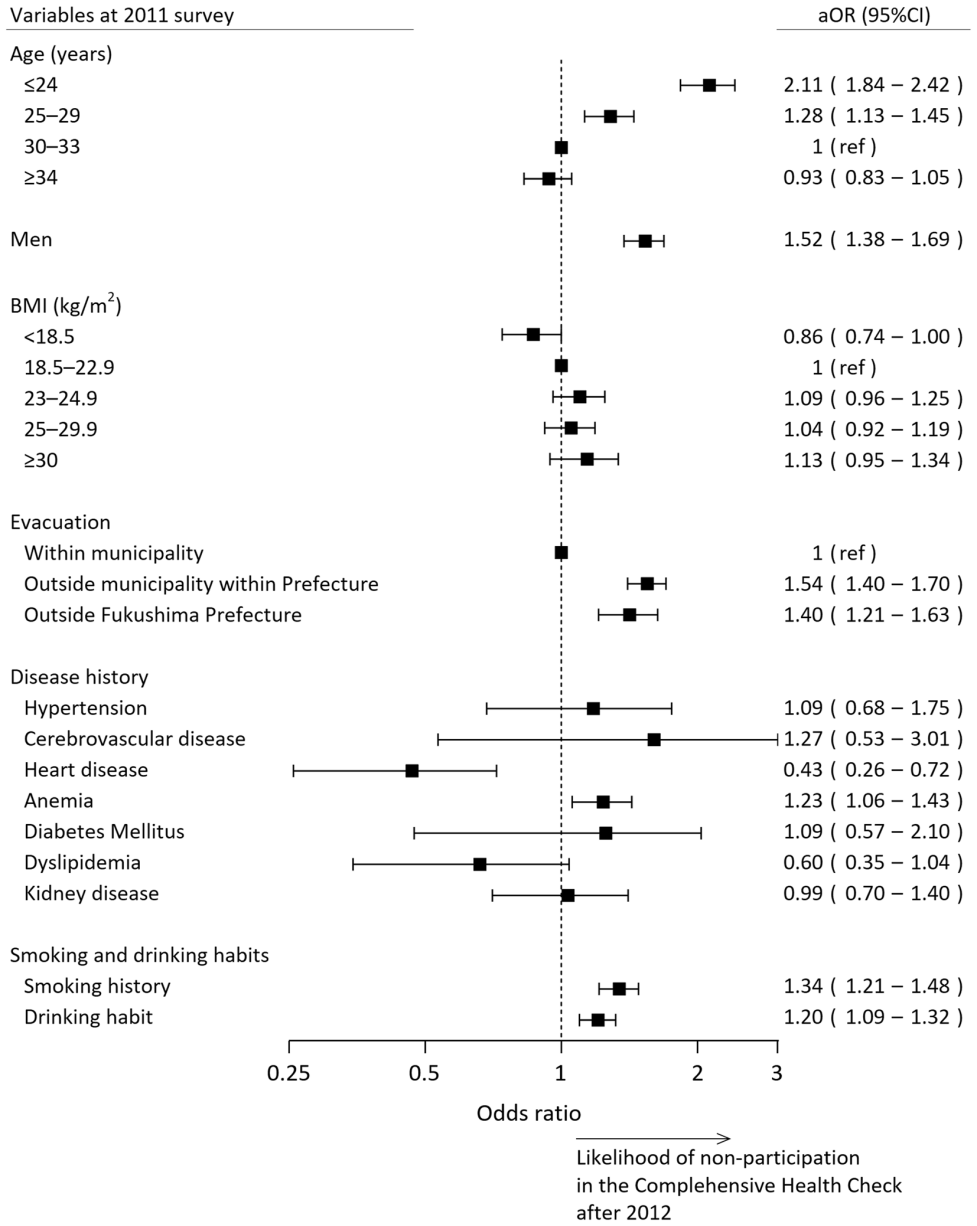
Multivariate analysis to predict non-participation in the Comprehensive Health Check in the Fukushima Health Management Survey. A forest plot of the likelihood of non-participation in the Comprehensive Health Check after 2012 is shown. The higher odds indicate a higher likelihood of non-participation after the second survey. *aOR* adjusted odds ratio, *BMI* body mass index, *CI* confidence interval.

We examined the baseline characteristics associated with non-participation after the second survey in a multivariate logistic regression model. The multivariate analysis revealed the following variables as the independent predictors of the non-participation: age ≤ 24 years (adjusted odds ratio 2.11, 95% CI 1.84–2.42), 25–29 years of age (1.28, 1.13–1.45), men (1.52, 1.38–1.69), evacuation outside the municipality but within Fukushima prefecture (1.54, 1.40–1.70), evacuation outside the Fukushima prefecture (1.40, 1.21–1.63), medical history of heart disease (0.43, 0.26–0.72), anemia (1.23, 1.06–1.43), smoking habit (1.34, 1.21–1.48), and drinking habit (1.20, 1.09–1.32) (Fig. 2).

## Discussion

The FHMS is one of the largest disaster cohort studies in the world and has been conducted for more than a decade. This study revealed a substantial decline in the participation rate of the Comprehensive Health Check during the study period in the evacuees after the FDNPP accident who aged 20–37 years. Despite a large investment and cooperation of public bodies of national and prefectural government and a medical university, the participation rate of the FHMS has been low. The lowering participation rate in the disaster cohort survey may lead to study bias. Thus, efforts should be made to encourage the evacuees in the survey, and careful interpretation is necessary when discussing the study results.

A large reduction in the participation rate was observed between the itinial and second survey years in this study as 4029 (41%) of the 2011 participants did not participate in the second survey year of 2012 or later. Why did they discontinue the Comprehensive Health Check? The health anxiety of the evacuees might be associated with the change in the participation. Residents could not obtain accurate information on radiation doses immediately after the FDNPP accident, and they would have felt anxious about their health because of radiation. On account of the issuance of evacuation orders, many residents were forced to evacuate to temporary housing or other places of residence. The external and internal exposure surveys conducted immediately after the FDNPP accident revealed that the estimated median annual external dose was less than one mSv during the first 4 months between March and July 2011^23^. The internal exposure dose of 99.986% of approximately 184,000 residents was less than one mSv^24^. A study showed that radiation-related health concerns among evacuees were still high in 2020 compared to those who were not evacuated^25^. Another study found that radiation-related anxiety among Fukushima residents was resolved over time between 2012 and 2015^26^. It has been noted that people with higher awareness of radiation risks are more likely to participate in internal radiation exposure screening programs. Thus, residents whose health anxiety improved earlier may have discontinued participating in the Comprehensive Health Check.

This study found that younger age (≤ 29 years), men, evacuation outside the municipality, anemia, drinking, and smoking habits were related to discontinuation of participation in the Comprehensive Health Check. Whereas a history of heart disease were associated with continuous participation. Various studies have reported factors that affect the uptake of physical examinations. For instance, a survey identifying factors related to an unwillingness to participate in health examinations among adults aged 19 years and older showed that 9% were unwilling to participate^27^. Being men, smoking, having higher self-rated health, never been invited before, and not being willing to pay money were related to the unwillingness. An online survey of willingness to participate in secondary prevention programs among individuals aged 18–75 years reported that younger people, men, manual workers, unemployed people, and those who did not regularly practice physical activity were less likely to participate in health check programs^5^. This study is consistent with such previous studies. The anemia, which was identified as a contributor of non-participation, might be underrecognized in this cohort despite it has been a global health burden, including Japan^28^. Therefore, it is necessary to proactively educate people who are likely to not participate in secondary prevention programs and encourage them to attend health examinations.

The evacuation outside the municipality regardless within and outside of Fukushima prefecure was also accociated with non-participation after second survey. The evacuation zone after the FDNPP accident was revised multiple times, forcing evacuees to move to different areas frequently^29,30^. The evacuees have been invited for the Comprehensive Health Check by mail as described in the “Methods” section. Their new address will be easilty identified by the public office if they are within their home municipality, and they will receive the notification of the examination. However, they will not be notified unless they apply for their new addresses outside of the municipality. Combined with the confusion immediately after the earthquake, some evacuees who evacuated outside their home municipality might not know the opportunity of the Comprehensive Health Check. The remote evacuation might contribute to the low rate of participation in the first year of the program as extimated as 26.6%.

Some improvements can be made to minimize their non-participation. We found evacuees with younger ages ≤ 29 years as a predictor of the non-participation. Online communication may help to maintain their participation. Younger and more educated individuals may choose to respond online rather than in person^31,32^. There is a trend toward the practical application of digital technology in clinical trials that have been progressing during the coronavirus disease (COVID) era^33^. Financial incentives might encourage continued participation since participation in the FHMS is on a voluntary basis^34^.

While we believe that this study provides valuable information on post-disaster cohort studies, it has some limitations. First, it is not possible to generalize the results of this study. The FHMS is a disaster cohort study conducted after the Great East Japan Earthquake and the subsequent FDNPP accident. It is not clear whether the results can be replicated for other types of disasters or for disasters occurring in other regions. Second, there was no information on the characteristics of evacuees who did not participate in the entire series of the Comprehensive Health Check. This information cannot be identified unless the present data is combined with the data from health examinations conducted by municipal governments or workplaces. Existing results of health records by the health insurance system should be linked to the FHMS. It takes double effort for evacuees to participate in the Comprehensive Health Check of the FHMS and that of their health insurance system. A study on the reasons for non-participation in health examinations among 35–40 years old individuals found that the most common reasons were lack of time or interference with work^35^. The impact of evacuation due to the disaster can be investigated in greater detail by linking data from existing pre- and post-disaster medical records and the FHMS. A comparison of the data before and after the earthquake should be considered in the future to identify the populations vulnerable to the effects of major disasters.

## Conclusion

We found a lowering participation rate in the Comprehensive Health Check of the FHMS in the evacuees aged 20–37 years, especially among younger evacuees who were men, evacuated outside the municipality, and had histories of anemia, smoking, and drinking habits. We might have missed disaster-related health risks in such evacuees. Having a history of heart disease was associated with continued participation in the survey. It is necessary to consider measures to increase the participation rate in the Comprehensive Health Check of the FHMS.

## Supplementary Information

Supplementary Information.

## Data Availability

The datasets analyzed during the present study are not publicly available because the data from the Fukushima Health Management Survey belongs to the government of Fukushima Prefecture and can only be used within the organization.
