# Supplementary material for: Loss of participation among evacuees aged 20–37 years in the disaster cohort study after the Great East Japan Earthquake
Source: Sci Rep. 2022 Nov 15;12:19600. doi: 10.1038/s41598-022-23896-1 (PMC9665037; doi:10.1038/s41598-022-23896-1)
Supplement: Supplementary file 1 — Supplementary Information. [file 41598_2022_23896_MOESM1_ESM.docx]

# Supplemental material

# eTable 1. Estimation of the population eligible for the Comprehensive Health Check of the Fukushima Health Management Survey in March 2011.

# eFigure 1. The study population in the Comprehensive Health Check of the Fukushima Health Management Survey.

# eFigure 2. Changes in the Comprehensive Health Check of the Fukushima Health Management Survey, by gender and age group

# eTable 1. Estimation of the population eligible for the Comprehensive Health Check of the Fukushima Health Management Survey in March 2011.

| **Municipal district** | **Age group** | | | |
| --- | --- | --- | --- | --- |
|  | **20 – 24 yrs** | **25 – 29 yrs** | **30 – 34 yrs** | **35 – 37 yrs^a^** |
| Tamura City | 1,674 | 1,919 | 1,831 | 1,129 |
| Minami-Soma City | 2,479 | 3,322 | 4,157 | 2,788 |
| Kawamata Town | 555 | 695 | 744 | 486 |
| Hirono Town | 216 | 284 | 292 | 175 |
| Naraha Town | 308 | 332 | 419 | 246 |
| Tomioka Town | 708 | 950 | 1001 | 692 |
| Kawauchi Village | 94 | 128 | 126 | 64 |
| Ohkuma Town | 534 | 730 | 764 | 493 |
| Futaba Town | 271 | 347 | 392 | 241 |
| Namie Town | 761 | 1,094 | 1,136 | 667 |
| Katsurao Village | 68 | 65 | 76 | 33 |
| Iitate Village | 209 | 295 | 264 | 178 |
| Part of Date City^b^ | 16 | 18 | 21 | 15 |

Data were obtained from the Fukushima Prefecture Population Survey conducted in March 2011 (https://www.pref.fukushima.lg.jp/sec/11045b/15859.html).

^a^The population between 35 and 37 years was estimated based on the population between 35 and 39 years old.

^b^A part of Date City was designated as an evacuation zone, where 413 people lived according to the 81st Disaster Control Meeting of the Date City on July 1, 2011. (The meeting minutes are available at https://www.city.fukushima-date.lg.jp/uploaded/attachment/1664.pdf). We estimated the population in the evacuation zone in Date City using the population of the same city classified by age group.

# eFigure 1. Study population in the Comprehensive Health Check of the Fukushima Health Management Survey.


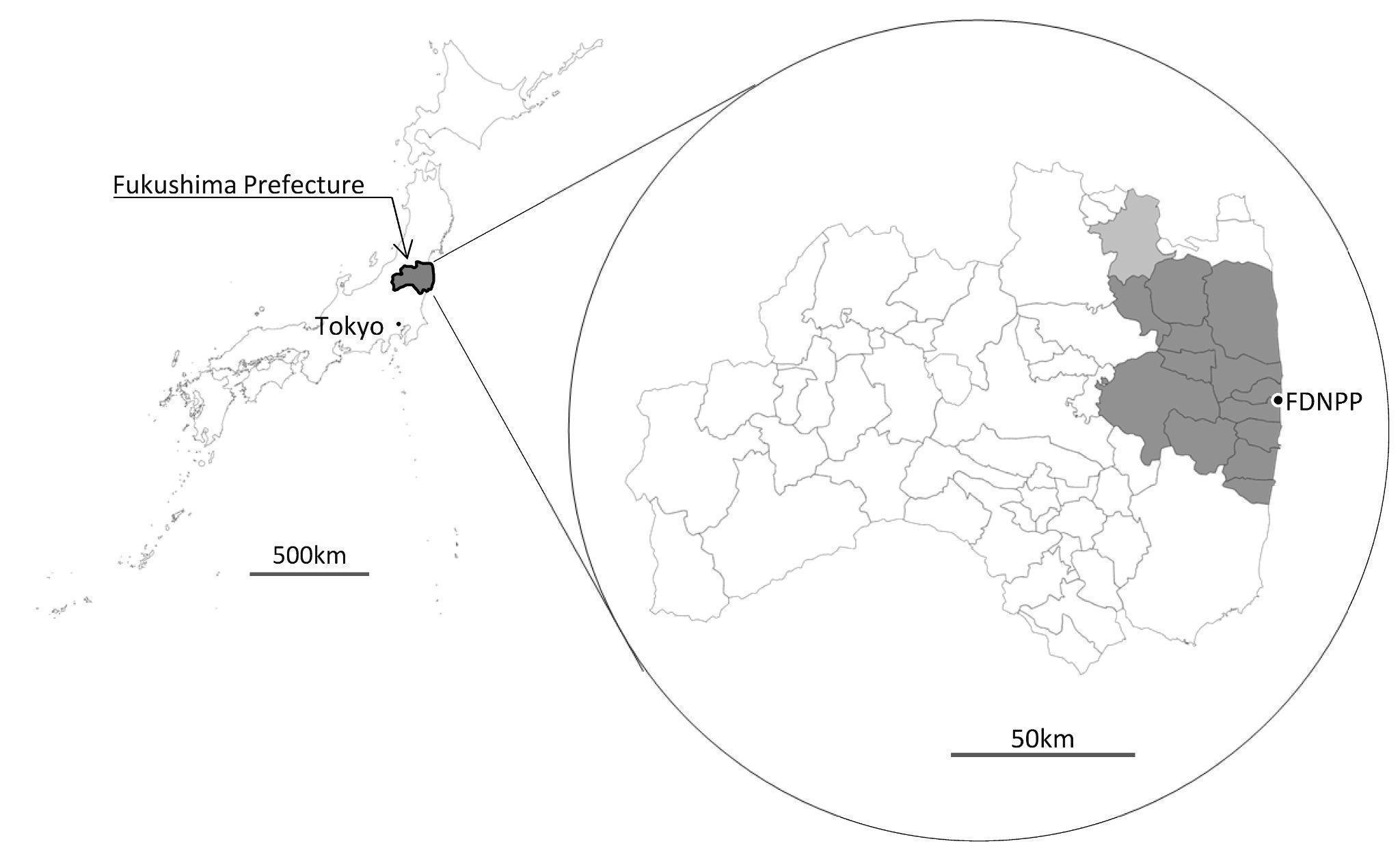


The cohort of Comprehensive Health Check of the Fukushima Health Management Survey is shown geographically. Residents in the municipalities around the Fukushima Daiichi Nuclear Power Plant (FDNPP), consisting of Tamura and Minami-soma cities, and Kawamata, Hirono, Naraha, Tomioka, Kawauchi, Ohkuma, Futaba, Namie, Katsurao, and Iitate villages (dark areas in the map), participated in the health checkup. Residents in a part of Date City (gray area) were also eligible for the Comprehensive Health Check.

The figure was created using the blank map publicly available from Geospatial Information Authority of Japan (https://maps.gsi.go.jp/) under a CC BY open access license (http://creativecommons.org/licenses/by/4.0/) and modified by M.Takita using Microsoft PowerPoint version 2202 (Microsoft Corporation).

# eFigure 2. Changes in the Comprehensive Health Check of the Fukushima Health Management Survey, by gender and age group


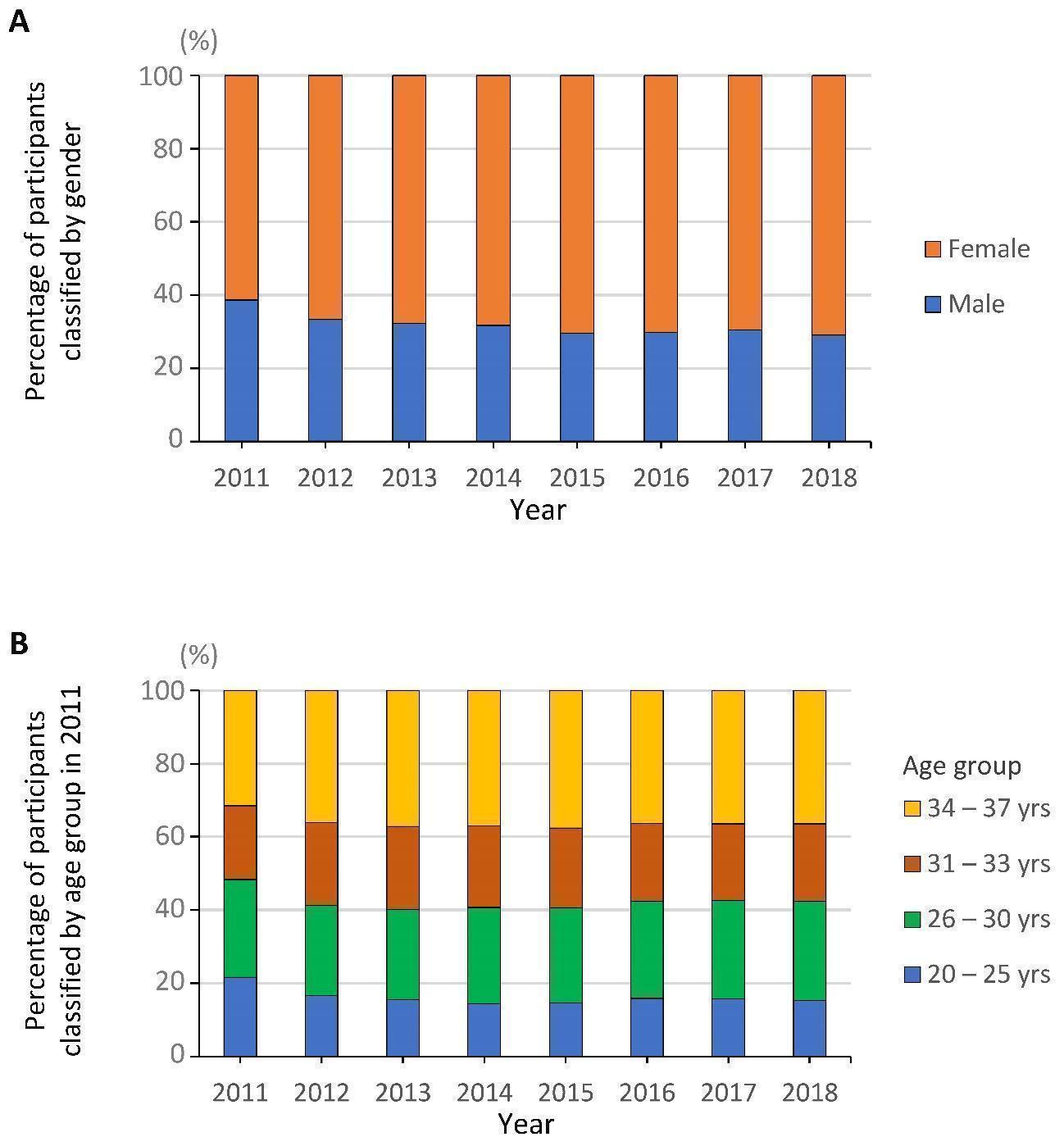


The annual changes classified by gender (A) and age group in the 2011 survey (B) are shown for the Comprehensive Health Check of the Fukushima Health Management Survey.
